# Induced Native Phage Therapy for the Treatment of Lyme Disease and Relapsing Fever: A Retrospective Review of First 14 Months in One Clinic

**DOI:** 10.7759/cureus.20014

**Published:** 2021-11-29

**Authors:** David A Jernigan, Martin C Hart, Keeley K Dodd, Samuel Jameson, Todd Farney

**Affiliations:** 1 Biological Medicine, Biologix Center for Optimum Health, Franklin, USA; 2 Research and Development, PhagenCorp, LLC, Sarasota, USA

**Keywords:** borrelia, bacteriophage therapy, herxheimer reaction, post-treatment lyme disease syndrome (ptlds), relapsing fever, chronic lyme disease, inducen-ld/rf, induced native phage therapy (inpt)

## Abstract

The overall failure rate of standard therapeutic options for late/chronic/persistent borreliosis emphasizes the need for novel therapeutic strategies. In this report, we are presenting a novel therapeutic option based on a new technology, Induced Native Phage Therapy (INPT; PhagenCorp, LLC, Sarasota, FL), and its ability to facilitate the elimination of infection more rapidly, efficiently, and with less harm to the patient than conventional treatments.

*Borrelia* species in the environment are themselves always infected by their own type of *Borrelia* bacteriophages. Both the *Borrelia* spirochete and the *Borrelia* bacteriophages are transmitted into humans via the bite of a vector, such as ticks. The *Borrelia* bacteriophages (phages) are called native phages in that they coexist naturally within the human body, and only infect the specific bacteria host population. Native phages persist in humans only as long as there are host bacteria of the correct type to continue replicating more phages. The purposeful manipulation of native phages to kill their host bacteria is the basis of INPT. INPT is a patent-pending technology that uses a proprietary adjunctive assay called Biospectral Emission Sequencing to identify and isolate the specific complex electromagnetic signatures necessary to induce the native phages to epigenetically revert from their normal quiescent, lysogenic activity to virulent, lytic activity, thereby killing their host bacteria. The strategic subtle, low-frequency/low-energy signatures are imprinted into a proprietary oral formula, Inducen-LD, which serves as a carrier to introduce the signals therapeutically into the body.

As a proof-of-concept method validation, a total of 26 patients with post-treatment (antibiotic) Lyme disease syndrome, who initially were found upon Phelix Borrelia-phage testing (R.E.D. Laboratories, Belgium) to have one or more *Borrelia* species, were submitted to INPT treatment. A total of 20 patients (77%) were found to be negative after two weeks of the total program of care. Six patients who remained positive after the initial therapy received an extended INPT treatment and were retested. Four were subsequently found to be negative for one or more of their previously diagnosed *Borrelia* strains. Thus a total of 24 out of 26 (92%) patients were successfully treated with INPT. Mild to substantial clinical improvements were reported by all participants without noticeable adverse reactions to the INPT treatments. We have demonstrated a possible mechanism in which native bacteriophages can be induced to epigenetically switch from lysogenic to lytic actions, thereby eliminating the targeted bacteria efficiently, with little to no harm to tissues or the microbiome.

## Introduction

Lyme disease (LD) is caused by a spirochete infection of *Borrelia burgdorferi, Borrelia garinii, and Borrelia* *afzelii,* but these are just a few of the 20 strains of *Borrelia* that can be transmitted by blood-sucking insects, such as ticks and mosquitos. Relapsing fever (RF) is caused by several different species of *Borrelia*, such as *Borrelia miyamotoi*,* Borrelia hermsii, Borrelia parkeri, *and *Borrelia turicatae*. In this review, all but one patient had indications of one or more of the bacteria in the RF group, with the one patient having indications of the *Borrelia burgdorferi sensu lato* associated with LD. The authors note, as have previous researchers, that patients with *B. miyamotoi* seem to have more severe symptoms and may be more antibiotic-resistant leading to the chronic nature of their illness [1].

Current treatments for LD and RF have divided medical opinion with debates over the long-term use of aggressive antibiotic treatments employed by some practitioners, the side effects and often severe Herxheimer reactions of which can be extremely difficult for the already infection-challenged patient to tolerate [2]. Based upon our review of cases from around the United States, the disagreements over the use of long-term antibiotics may be due to the incorrect diagnosis of LD, when it is truly RF, a condition that often does not respond to the same antibiotics as do the bacteria of LD. The newest and most sensitive laboratory testing for LD and RF reveals that many people do not continue to suffer after completing prolonged antibiotic therapy from illness due to a post-treatment Lyme disease syndrome (PTLDS), but due to persistent infection. Relapses in symptoms are common due to the inefficiency of antibiotics to kill all the target populations of bacteria [3].

David Kaufman, MD and Ilene Ruhoy, MD, Ph.D. clarify an important fact to consider in the treatment of any single infection in cases of chronic LD and the achievement of restoring the patient to a state of being symptom-free. Kaufman and Ruhoy state, “Chronic Lyme disease (CLD) is a complex chronic illness. Controversy exists regarding whether it represents persistent Lyme infection or a post-infectious, possibly autoimmune syndrome, or a combination of both. This is an important topic as a greater understanding of CLD can help guide treatment options for these patients who suffer sometimes for decades and are often turned away from healthcare providers” [4]. With the advent of the Phelix Borrelia-phage (PBP) testing, we can now determine the presence or absence of *Borrelia* infection with a much higher degree of certainty. The review we are presenting here is regarding a new technology, Induced Native Phage Therapy (INPT; PhagenCorp, LLC, Sarasota, FL), and its ability to facilitate the elimination of infection more rapidly, efficiently, and with less harm to the patient than conventional treatments. The premise behind the technology of INPT and the treatment based upon this technology, Inducen-LD/RF, is similar to other peer-reviewed, emerging technologies, which present the therapeutic effects of biogenic, low-level electromagnetic (EM) frequencies influencing epigenetic changes in targeted substances in the treatment of cancer, as well as the inactivation of viruses using pulsed visible light [5,6]. Native bacteriophages are those found already existing within the patient’s body, having entered the body with the acquired bacterial infection. Restoring health in totality in the severely affected patient requires addressing the total condition of the patient, beyond the infection, which is why the patients in this review underwent treatment with the Inducen-LD/RF as part of our standard program of daily outpatient holistic care for the one to four weeks of treatment.

Bacteriophages (phages) are known to be ubiquitous in nature, outnumbering all other microbial life forms on the planet [7]. Forest Rowher, Ph.D., a microbial ecologist at San Diego State University, states that phages cause a trillion successful infections per second. Phages are viruses that only infect a specific host bacterium, which the phage uses to replicate more phages, a process called lysogenic activity. In the lysogenic process, the phage moves through the body, seeking its specific type of host bacterium. The phage lands on the surface of the bacteria and penetrates the bacterial membrane with a needle-like apparatus, injecting its genome into the bacteria, where it will be incorporated into the bacterial genetic engine, causing the bacteria to produce more phages.

The lysogenic process ultimately kills the host bacterium releasing prophages into the body to find another of their host bacteria to repeat the cycle [8]. This phage/bacteria parasitic relationship is ongoing across the entire globe regulating the size of every type of bacterial population. Phages are reported to kill an estimated 25-58% of all the various bacteria in the oceans and coastal regions every day as the result of their host/parasite lysogenic activities [9]. This lysogenic activity causes the bacteria to produce hundreds to several thousand prophages, the phage progeny, before the process kills the bacterium, releasing the prophages to seek out more of that type of bacteria to infect, whether in nature or a person’s body.

The bacteria/phage interactions have been ongoing from the beginning of time; therefore, it comes as no surprise that the bacteria found within vectors, such as ticks, are themselves always infected with their own phage-type. When the tick injects *Borrelia* into a person, the *Borrelia* are already infected with their unique type of phages native to that species of *Borrelia*. The native phages persist if there are host bacteria of the correct type to continue replicating more phages. It is estimated that native phages are much more prevalent in a person than the actual bacteria by a factor of 10 times [10]. Bacteriophages are the most abundant viruses in the body [11]. It is the prevalence of phages that has led to the development of extremely sensitive laboratory phage quantitative polymerase chain reaction (qPCR) testing, the PBP test, which was utilized for our study [12].

The diagnosis of *Borrelia* infections has been historically very poor with low sensitivity and low reliability [13]. To make matters worse, the various strains of *Borrelia*, such as those associated with LD and RF, are morphologically similar and clinically present with almost indistinguishable symptoms. The PBP testing brings a much higher level of diagnostic and treatment confidence with its ability to identify the exact strain of *Borrelia*, whether early or late in the disease progression [12]. Treatment selection can be tailored to the specific type of infection, a very important fact since LD and RF require different medications. Each species of *Borrelia* is host to a specific phage. *B. burgdorferi* bacterium is infected by *B. burgdorferi *phages, *B. miyamotoi* bacteria by *B. miyamotoi *phages, and so on. The PBP testing can identify the genetics of each type of *Borrelia *phage to determine exactly what type of *Borrelia* bacteria is present in the patient. INPT's specificity to the target bacteria ensures the treatment has no adverse effects on the microbiome flora, in contrast to conventional broad-spectrum interventions.

According to Finzi et al., lysogeny is very stable, and yet, the switch to lysis is very efficient [14]. It is the nature of native phages to propagate quiescently, via lysogenic activity. At times, due to previously poorly understood environmental causes, the phages can suddenly switch from quiescent, lysogenic activities to virulent, lytic swarming, killing all their host bacterial population. In previously reported conventional bacteriophage therapy, the lysis of the target bacterial infection was so rapid that, at times, it was reported to have a therapeutic effect within four to five hours, and cure being pronounced within 24-48 hours [15]. In some bacteriophage types, this epigenetic switch to the virulent, lytic mode, is irreversible, leading to the complete annihilation of the host bacteria [14]. Native phages can penetrate tissues, cells, biofilms, and cross the blood-brain barrier to kill each of their host bacteria [16]. The purposeful manipulation of native phages to kill their host bacteria is the basis of INPT.

INPT is a patent-pending technology that utilizes a proprietary, adjunctive testing technology called Biospectral Emission Sequencing (BES) to identify and isolate the specific complex electromagnetic signatures (EMS) required to imprint into the Inducen-LD/RF to stimulate or induce epigenetic alterations exclusively in the specific type of bacteriophages that use the target bacteria as a host. INPT does not seek to treat the bacteria, as in the case of bactericidal or bacteriostatic antibiotics, but to use subtle low-frequency/low-energy to activate native phage activity causing them to kill the infection. In a similar study, it is reported that subtle, low-frequency/low-energy environmental changes, such as exposure of the person to ambient (subtle frequencies) 50 Hz EM fields, can induce Epstein-Barr virus from a quiescent or lysogenic mode to the virulent or lytic mode [17]. It is believed that the subtle frequencies of INPT are sequenced in such a way as to induce only the specific type of native phage that will target the bacterial infection, causing those quiescent native phages to epigenetically switch to lytic mode, thereby eliminating the infection. Epigenetic gene regulation is where gene expression is modified without involving changes in the phage DNA sequence [18]. In causing the lytic effect, the specific triggering proprietary sequences of EMS imprinted into the INPT treatment, Inducen-LD/RF, are believed to cause redirection of the cellular expression of the phages by either indirect epigenetic regulation, where cellular signaling or transcriptional dysregulation occurs, or direct epigenetic regulation, where epigenetic cofactors such as histone deacetylases are targeted. In viruses/phages, transformation is a consequence of the expression of the viral latency proteins and RNAs, which again can have either a direct or indirect effect on epigenetic regulation of cellular expression [19]. More research is needed to clarify the exact mechanism of the Inducen-LD/RF treatment. This review is observing a direct and strategic input and its effect and presents possible mechanisms of action.

The Inducen-LD/RF is in 2-ml glass ampules for oral use, containing a sterile formulation of distilled water, salt, and Cinis Equiseti (mineral ash of horsetail herb), the latter of which is diluted to one part per million. The mineral content per dose is of minimal biological effect and serves only to enhance the stability of the imprinted low-frequency/low-energy EMS. The liquid formulation serves as a carrier substance for the EMS. When introduced to the body orally, Inducen-LD/RF is observed, via adjunctive testing, to broadcast the induction frequencies, virtually instantaneously throughout the liquid-crystalline matrix to all parts of the body with little to no perceptible tissue impedance. Subtle inputs into a biological system have been previously documented to be significantly augmented via biological signal amplification [20]. Tissue signal amplification may explain the observed global effect of the low-frequency/low-energy fields required to induce epigenetic changes in the targeted phages to revert from quiescent lysogenic activity to lytic swarming of their host bacterium [5]. The speed of activation of the lytic killing of their host bacterium appears instantaneous and continues with sustained treatment until the last host bacterium is dead.

In summary, this study demonstrates a proposed mechanism in which INPT can induce native phages to switch from lysogenic activities to lytic swarming to rapidly eliminate the targeted strains of *Borrelia*, within two weeks as verified by the disappearance of their native phages and subsequent clinical improvements. We have also demonstrated for the first time in *Borrelia*-related treatment that it is possible to work with a heretofore unexplored aspect of the body’s natural antimicrobial defense, native phages, in the fight against LD and RF bacteria. It appears the infection of our infection can be favorably manipulated for the elimination of the cause of the disease.

## Materials and methods

A single-institution, IRB-approved, retrospective review of patients with treatment-resistant illness, diagnosed between 2020 and 2021, was performed. Descriptive statistics of the laboratory findings and treatment characteristics were performed.

Patient selection

The inclusion criteria were as follows: history of previously diagnosed LD, with persistent or recurring symptoms beyond antibiotic interventions; history of previously diagnosed LD with failure of antibiotic treatment; and confirmation of the presence of any of the 20 species of *Borrelia *phages upon PBP qPCR testing. There were no exclusion criteria.

Phelix Borrelia-phage testing

No published normative data existed for the use of INPT in patients with LD or RF in that it is an emerging technology; however, prior peer-reviewed technologies support the premise [21]. The clinical advancements of the technologies leading up to the development of INPT took place over the course of 26 years, the effects of which were largely restricted to clinical observation and adjunctive BES testing incorporated into the standard programs of care, until the introduction of the newly released laboratory test, PBP qPCR [22]. High-sensitivity of >90% and 100% specificity, showing no false positives and few false negatives, combined with the speed of verification of treatment effect being only four days post-bacterial clearance, made the PBP testing ideal for detection of early or late LD/RF.

Conventional antibody testing, such as Lyme Western blot testing would not be of use because antibodies remain circulating in the body for years after the infection is gone, and the testing presents a lack of sensitivity, especially in late-stage illness [23]. The limitations of immunoglobulin M/immunoglobulin G antibody testing and the need for more sensitive testing for *Borrelia* species are well documented [24]. Discussions of any current assay being able to determine an active or inactive infection are not relevant to this review. More important to this review is the apparent frank presence or absence of the infection as indicated by the presence or absence of the bacteria’s native phages. This criterion is achieved only by the PBP test.

The PBP test undergoes quadruplicate real-time polymerase chain reaction (PCR) tests for three different targets (*B. burgdorferi sl*, *B. miyamotoi*, and RF group) for a total of 12 assessments. All positive-like samples are submitted to confirmatory sequencing to rule out false-positive results. The Phelix Phage test (patent no. WO2018083491A1) is performed on whole blood from two ethylenediaminetetraacetic acid (EDTA) tubes. The very first step consists of extracting the DNA using a specific manual method to ensure the best possible recovery of the pathogenic DNA. The extracted DNA undergoes three different qPCRs using proprietary primers and probes for phage-specific detection. The three qPCRs aim to detect the following targets (i) *Borrelia miyamotoi*, (ii)* Borrelia burgdorferi sensu lato* (*B. burgdorferi ss, B. bissettii, B. bavariensis, B. valaisiana, B. afzelii, and B. garinii)*, and (iii) RF (*B. hermsii, B. recurrentis, B. crocidurae, B. duttonii*). Each target is tested in a quadruplicate. The amplified fragments are then analyzed by sequencing to confirm the positivity of the sample (i.e., to rule out false positives).

INPT (Inducen-LD/RF) treatment

In successful treatment, eliminating the cause, i.e. the bacteria, does not always eliminate the tissue damage, system deregulation, and symptoms from the prolonged infection, especially in long-standing, multi-system illnesses, such as LD and RF [25]. The primary goal of any treatment is to remove the offending agent/s causing the illness with as minimal secondary harm to the body and mind as possible, simultaneously facilitating the restoration of health until the desired quality of life is achieved. Our use of INPT via Inducen-LD/RF in combination with the program of comprehensive care was designed to work in alignment with natural processes in the body, addressing both the cause and the effect to improve the quality of life.

The selection of the dosage of Inducen-LD/RF was set at 2 ml, twice per day for five days for all patients. This dosage was based upon prior adjunctive BES testing of the required threshold energy of activation of the targeted phages. It was previously determined via clinical observation and adjunctive BES testing, that the energy of activation faded over the course of the day and required a second dose in the evening. It was not determined if one larger dose would carry through to the next day, although prior observation of larger doses did not appear to prove more beneficial, nor did it negate the need for a second evening dose. Dosages of 2 ml, three to five times per day have been done with no reported or observed adverse issues in prior patients. Compliance with all recommended Inducen dosages was discussed with all participants to avoid desensitizing of the phages through sporadically taking their recommended dosages; the desire being to keep the phages activated to lysis against their natural state of lysogeny. To determine the effectiveness of INPT as a monotherapy, no other anti-infective remedies were given, except for eight patients who were given one or more low-dose, bacteria-directed immune support products (*Juglans nigra*, *Scutellaria baicalensis*, and *Cryptolepis sanguinolenta*) in combination with their Inducen-LD/RF treatment to rule out the potential for interference with the action of the Inducen-LD/RF. The use of these botanicals in conjunction with the Inducen-LD/RF demonstrates no interference occurred when other modalities were added. The lack of interference is an important finding since many chronically ill patients self-medicate with a variety of antimicrobial supplements. These eight patients were included in the negative PBP testing category, due to the inability of those immune-boosting products to result in a negative test result within the one to four-week period of time, the effect being attributed to the Inducen-LD/RF.

Patients and their families and/or significant other(s) were informed about potential risks, potential benefits, and the considerable uncertainties involved in the application of INPT, and this was amply documented in the patient record and posted educational materials. Patients were required to sign an informed consent document. It was routine to receive written permission from the patient if they desired us to communicate with the patient’s primary care physician and other healthcare professionals in advance or during their program of care so that they could be informed of the principles behind what we were doing and provide them with the opportunity to voice any concerns. Communication with the patient’s healthcare team when requested was also important to solicit cooperation in the management of any unforeseen event in the ongoing care of the patient. No treatments were added by external, co-managing physicians during the review period.

Each patient had a monthly follow-up in person, by email, or by telephone to monitor their clinical status, review surveillance laboratory results, concurrently recommend protocol modifications and any supplements, or the need for allied practitioners to provide support nearer to the patient’s home.

## Results

There were 19 females and seven males, ranging from 17 to 65 years old. All 26 patients were previously diagnosed with *Borrelia* infection and treated extensively with conventional antibiotics, yet had remained symptomatic. Due to the suspicion of persistent post-treatment *Borrelia* infection, all patients were tested using PBP blood tests. All 26 patients were tested and confirmed as still positive, despite previous antibiotic treatment, for one or more species of *Borrelia* via PBP qPCR lab testing. In the group of 26 patients, 52% (14 people) had *Borrelia miyamotoi* detected, which is considered in the RF group but is clinically distinct, resembling severe cases of persistent LD in its presentation, only more treatment-resistant and at times more severe [1]. Of the patients, 61% (16 people) were found to have *Borrelia* phage strains that fall under the category of RF group from one or more of the following strains of *Borrelia*: *B. hermsii*, *B. recurrentis*, *B. crocidurae*, and *B. duttonii*. One person in the group had *B. burgdorferi sensu lato*.

The PBP qPCR test was utilized as our standard due to its very high sensitivity to detecting the presence of infection and the immediate evidence of the absence of infection post-treatment. As presented by Louis Teulieres, MD, at the 2019 International Lyme and Associated Diseases Society conference, the PBP testing is statistically the most accurate test.

It is important to note that no externally derived bacteriophages are used in the Inducen-LD/RF formulation. Native bacteriophages enter the body upon the initial bacterial infection and therefore already exist within the patient [11]. INPT is believed to work by inducing native phages by epigenetic changes to revert from quiescent (lysogenic) activity to exert lytic action on their host, with the goal of the complete elimination of the target bacteria. INPT-based treatments, such as Inducen-LD/RF, hold the promise to be very safe treatments due to their very high specificity to the targeted bacteriophage, and that phage’s very high specificity to its host bacteria.

All 26 patients received one five-day course of treatment with Inducen-LD/RF at a consistent daily dosage of 2 ml in the morning and evening, as part of our standard, one to four-week, daily holistic program of care. No other anti-infective agents were used by any of the patients, except for eight patients who took one or more, low-dose, bacteria-directed immune support (*Juglans nigra*, *Scutellaria baicalensis*, and *Cryptolepis sanguinolenta*) in combination with their Inducen-LD/RF as an interference challenge, that were included in the negative category, due to the unlikely ability of those products to result in a negative test result within the one to four-week program in the chronically-ill patients in this review. Future studies of concomitant use of Inducen-LD/RF with antibiotics and/or botanicals are warranted. Documenting clinical/symptom improvements is not the primary focus of this review due to the short time span between the implementation of the Inducen-LD/RF treatment on day two of their one to four-week program and cessation of the Inducen-LD/RF on day six of the program. Clinical improvements were objectively and subjectively monitored; however, due to the nature of the chronic and inflammatory conditions that all patients had suffered for years, and the resulting tissue and system damage, the primary goal of this retrospective review was to document the apparent rapid elimination of the specific *Borrelia* species with which each person was found to be infected. All 26 patients adjunctively tested clear of *Borrelia* infection within three to five days of beginning their Inducen-LD/RF treatment. According to research presented by Louis Teulieres, MD, at the 2019 International Lyme and Associated Diseases conference, phages will die within four days of the death of the last host bacteria due to the phage’s inability to readily find suitable alternative hosts to replicate themselves. All the patients were tested again on day 11 or beyond, via PBP qPCR to allow at least six days for the native phages to die.

In total, 20 out of 26 (77%) initially positive patients were found to be negative for one or more of their previously diagnosed strains of *Borrelia* after a five-day treatment with Inducen-LD/RF. Post-treatment confirmatory PBP testing was performed between six and 30 days after completion of the five days of INPT treatment. In the 20 negative patients, eight (40%) also took low-dose, bacteria-directed immune support (*Juglans nigra*, *Scutellaria baicalensis*, and *Cryptolepis sanguinolenta*) in combination with their Inducen-LD/RF. The use of these botanicals in conjunction with the Inducen-LD/RF demonstrated that no interference occurred when other modalities were added. These people were included in the negative PBP testing category, due to the inability of those immune-boosting products to result in a negative test result within the one to four-week period of time, the effect being attributed to the Inducen-LD/RF. Of the six (23%) patients whom the Inducen-LD treatment failed, four received extended Inducen-LD treatment for up to 10 days, after which they were retested with PBP testing and found to be negative for one or more of their previously diagnosed *Borrelia* strains. The remaining two were lost to further follow-up. Adding these four patients to the 20 previous negative patients brings an increased total of 24 out of 26 (92%) successfully treated with INPT. To document long-term elimination of the infection in the 20 patients who tested as negative, eight were tested a third time, between eight and 24 weeks after their negative test, without receiving further Inducen-LD or antibiotic treatment. Of these eight patients, seven (88%) remained negative for the indications of their initial strain of *Borrelia*, demonstrating long-term clearance of the *Borrelia* without rebound growth of infection below the sensitivity of the testing to detect. However, three of the eight (38%) patients were found to have indications of a new strain of *Borrelia* suggesting a newly acquired infection, or a low-level, undetected infection, which took dominance in the absence of the initially treated strain. Of note, the one case of infection with *Borrelia burgdorferi sensu lato* was in the group of eight patients who remained negative up to 24 weeks when he was retested using PBP testing.

All patients reported mild to substantial clinical improvements including but not limited to decreased pain, decreased anxiety/depression, improved joint mobility and gait, improved neurological coordination, and improved mental clarity and muscle strength. One of the most impressive improvements was the ability of one patient to no longer need a wheelchair, which has continued for over one year post-treatment. It is the author’s opinion that these improvements are from the combined effect of the successful elimination of the targeted bacteria by the Inducen-LD/RF and the comprehensive holistic program of individualized care. As an emerging technology, much has been and will be learned to enhance future INPT treatment; however, we have observed that it has enabled more rapid improvements than previously used methods.

One of the hindrances to the success of INPT treatments is the defenses bacteria have developed over the millennia against attacks from phages. It is known that bacteria have potent anti-phage mechanisms, such as the CRISPR-Cas enzymes, retrons, and biofilms, although lytic phages have been shown to penetrate biofilms to reach their host bacteria [26,27]. Although phages are known to be able to penetrate through biofilms, the use of systemic proteolytic enzymes can help break down these biofilms, enabling phages to be more effective. A possible reason for treatment failure in six of the patients in the program is bacterial mutation due to long-term pressure from antibiotics changing the bacteria/phage dynamic [28]. In these cases, it could be beneficial to combine strategic prescriptive or natural antibacterial treatments with INPT, as is often done with conventional bacteriophage treatment. Future research may also lead to enhancements on INPT to enable phages to defeat these bacterial mutations and defense mechanisms, as well as possibly be able to enhance conventional bacteriophage therapies to attack the bacteria more directly and predictably.

Concerns of severe Jarisch-Herxheimer reactions

Consideration was given to the fact that if successful, killing all the *Borrelia* population in five days would cause a severe and potentially life-threatening Jarisch-Herxheimer reaction (JHR) from the release of what was believed to be endo and exotoxins. However, the toxin premise of JHR in LD treatment has been disproven. Cruz et al. report, “Genomic sequencing of *Borrelia burgdorferi* has revealed it lacks orthologs of exotoxins as well as the specialized machinery required to deliver noxious molecules into host cells” [29,30]. Takayama also confirmed that no endotoxins could be found in Lyme spirochetes [31]. According to Thomas Butler, the mechanism of JHR in spirochetal infections is due to the inefficient killing of the bacteria by antibiotics, enabling whole live bacteria to be engulfed by the body’s macrophages, causing the production of excessive amounts of proinflammatory cytokines, such as tumor necrosis factor (TNF), interleukin 6, and interleukin 8, within the macrophages [32]. The proinflammatory cytokine, histamine, is often increased in LD and RF patients, which may explain the common symptom of hypersensitivity reactions and intolerance to medications and environmental triggers in this patient population [33]. Conversely, according to Butler, when native phages lyse bacteria, there is no increased phagocytosis of live bacteria by macrophages, because lytic phages destroy the bacteria very rapidly before the macrophages can engulf any live bacteria, therefore they cannot create inflammation due to a cytokine storm, which may explain why all 26 patients tolerated the INPT treatment very well. Initial extensive lab baseline testing, including but more extensive than a standard complete blood count (CBC) and multi-chem, was performed prior to beginning the program. All post-program hematological testing demonstrated multiple improvements for participants as a result of the combined INPT and comprehensive, holistic, individualized program of care. Although very little worsening of symptoms was reported with the use of INPT, our observations outside of this review of combining INPT with natural or prescriptive antibacterial treatments once again saw increased worsening of symptoms.

Managing patient expectations with INPT

Managing patient expectations was one of the most challenging aspects observed in the apparent success of the treatment, documented by the post-treatment PBP lab testing. The belief that eliminating the infection should immediately eliminate the symptoms is deeply ingrained in the patient psyche. Achieving rapid and lasting restoration of health is to be expected in the case of infections that are identified and treated quickly before the infection can cause excessive tissue damage and significant autonomic dysregulation. Rapid restoration of health is less likely in long-standing infection, which has caused tissue damage and global system dysregulation. All of the patients in this review were in this latter category. Education of the patient to this fact is important prior to implementing any successful treatment of the infection. Comprehensive treatments which address the unique global disturbances in each patient are critical for achieving the desired quality of life.

## Discussion

We report on 26 patients that were identified over 14 months from 18 March 2020 to 18 May 2021 as having one or more strains of *Borrelia* and who received a five-day treatment of Inducen-LD/RF as part of our standard program of care. The initial data were gathered during a one to four-week program of comprehensive holistic care at a multi-doctor, independent practice in the United States of America. The use of electromagnetic therapies that seek to work with the natural design and function of the body, as well as towards the restoration of the integrity of the body in its totality, is within the discretion of the practitioners and is not considered “experimental” nor does it requires Institutional Review Board's (IRB) approval (Agency for Healthcare Quality and Research). This review, however, was conducted as a single-institution, IRB-approved, retrospective review of patients with treatment-resistant illness undergoing our standard program of care of which INPT is part.

Stimulating native phages for therapeutic effect is not to be considered as pertaining to conventional bacteriophage therapies, which instead seek to find, isolate, and introduce externally-derived phages into the diseased patient in the hope that they will kill the target infection. At this time, there are no conventional bacteriophage therapies identified to address LD or RF bacteria.

Although formal research protocols and randomized controlled trials (RCTs) play an important role in clinical research, there is the value of real-world evidence (RWE) and real-world data (RWD) as obtained through retrospective reviews and observational clinical studies. RCTs have been considered as more applicable and useful to large vertically integrated healthcare systems equipped with electronic medical records, but they acknowledge this may not be suitable for all situations, such as independent clinical studies [34]. Our retrospective review may be considered akin to a collection of “n-of-1” reports since each of our subjects had a track record of their courses using conventional antibiotics and often over very extended time periods [35]. These patients chose to pursue treatment with INPT given the limited effectiveness of their prior treatment and/or relapses after possibly having derived little or no benefit from conventional antibiotics or alternative modalities.

As for the mechanism of action in which INPT induces native *Borrelia *phages, the hypothesis has never been attempted; therefore, we are limited to observing the retrospective cause and effect. To achieve a similar outcome in the short treatment time frame and with such good treatment tolerance using conventional antibiotics has not been possible, with its well-recognized poor treatment tolerance, adverse effects, and prolonged treatment time frame to yield beneficial outcomes [2].

The non-toxic and non-allergenic nature of INPT (Inducen-LD/RF), combined with its extremely low energy inputs to the body, makes it an excellent treatment modality for extremely sensitive patients common to chronic or treatment-resistant LD and RF. The use of INPT for co-infections of LD and RF is beyond the scope of this paper; however, the same concepts apply to any microbial infection. Each microbe has its own native phage that uses it as a host [36]. INPT is tailored to target the specific species of phage for the specific species of bacteria infecting the patient. The INPT formula will not be able to stimulate a phage-type that does not exist within the patient; therefore, the inducing EM signature of that specific species in the formulation of Inducen-LD/RF will have a null effect on the patient, since it only acts on the phages, and not the body or immune system. Often a patient has more than one species of *Borrelia* [37]. All *Borrelia *species identified with testing can be targeted with specific, subtle EM signatures in the Inducen-LD/RF formula.

There is no conferred immunity to reinfection after successful treatment with the Inducen-LD/RF due to the fact that the native *Borrelia *phages die within four days of the last *Borrelia* bacteria dying. In the case of reinfection from another vector-borne infection, each new *Borrelia* infection enters the body already infected with its native phages. These phages can also be induced to lysis, having never been treated with the Inducen-LD/RF. There is no residual or perpetuated energy of activation of the Inducen-LD/RF treatment beyond the discontinuance of the treatment.

Conventional infection interventions most often seek and track the remission of symptoms, which according to the Cambridge Dictionary is “a period of time when an illness is less severe or is not affecting someone.” All treatment is done to decrease suffering and is a worthy goal; however, each treatment should be measured for its long-term risk-benefit comparison between the suffering caused by the treatment and the permanency of that treatment [38]. Due to the very high specificity of native phages to their host bacteria, Inducen-LD/RF appears to eliminate the infection based upon the understanding of the death of native phages, if there are no host bacteria present to continue replicating more phages. The sustained disappearance of *Borrelia *phages for as long as 26 weeks, as seen in this review, strongly suggests the complete elimination of the targeted infection [12]. With the rapid clearance of the infection by the native phages, we observed the typical clinical objectives for treatment rapidly shifted towards correcting the many consequences the infection inflicted upon the patient.

Although this review demonstrates the apparent proof of concept for INPT as part of a comprehensive program of holistic care, it was limited in the number of patients, due in most part to the travel fears and restrictions of coronavirus disease 2019 (COVID-19) preventing many people from participating. For this same reason, there were difficulties in achieving extensive and regular follow-up due to geographical distance, since most patients were traveling from other states and countries. COVID-19 also created difficulty in doing follow-up blood draws for confirmatory PBP testing during the period of the study. A more controlled, independent, grant-supported study is warranted with greater numbers of participants, who are only treated with Inducen-LD/RF with no comprehensive holistic program of care, as well as a study of patient demographics comprised of pre-and-post treatment, PBP-documented, newly infected individuals. In that the PBP testing only tests for *Borrelia* strains of infection at the time of this review, we could not document the many other types of potential tick-borne infections, using this sensitive form of bacteriophage laboratory testing.

INPT is a new class of what is called energy medicine, not being based upon homeopathy, radionics, or other recognized energetic modalities, and is believed to carry no risk. It is not directed at the body or treating the body. INPT is not treating diseased tissues, nor is it directly treating the bacterial infection, or seeking to modify the immune system or biochemistry of the body. INPT might be viewed in the same light as supplements that are used to boost the immune system to help the body fight infections, the difference being INPT is solely directed at modifying native phage activity within the targeted type of phage population and its innate predator/prey relationship with specific bacterial populations [39]. We observed no ancillary treatment interference, due to the totality of each patient's comprehensive individualized program of care to address their overall structural and functional integrity. There were no toxic or allergenic effects attributed to the Inducen-LD/RF reported by the group. It is our opinion that INPT is a safe and effective modality in the treatment of people with *Borrelia *infections. Treatment results may or may not be enhanced by the concomitant use of antibiotics or antibacterial botanical formulas, the results of which need to be studied further [40].

## Conclusions

It is our experience that INPT, specifically Inducen-LD/RF, as a monotherapy or as a concomitant therapy, is a viable treatment for people with LD and/or RF. During this review, it was important to document the minimum requirement of treatment duration and dosage to achieve the goal of confirming cause and effect in eliminating the infection and confirming it with the PBP test. Subsequent post-review patients have revealed a potential benefit of extending INPT treatment beyond the five-day period used in this program. Further study of INPT at independent facilities and institutions is warranted to clarify its mechanisms of action and to conduct formal treatment trials, as well as to improve upon what we have discovered.
